# The Unequal Effects of Social Distancing Policy on Subway Ridership during the COVID-19 Pandemic in Seoul, South Korea

**DOI:** 10.1007/s11524-021-00585-4

**Published:** 2022-01-01

**Authors:** Jaeyoung Ha, Suyoung Jo, Hee-kyoung Nam, Sung-il Cho

**Affiliations:** 1 Department of Public Health Science, Graduate School of Public Health, Seoul National University, 1 Gwanak-ro, Gwanak-gu, Seoul, 08826 Republic of Korea; 2 Institute of Health and Environment, Graduate School of Public Health, Seoul National University, Seoul, South Korea

## Abstract

In the Republic of Korea, social distancing policies relied on voluntary participation by citizens and exhibited short-term changes. In this situation, the effects of such policies varied depending on each community’s capacity to comply. Here, we collected subway ridership data for 294 stations on nine Seoul Metro lines and aggregated the data for each station to the 184 smallest administrative areas. We found that the mean percent change in subway ridership was fitted by an additive model of the log-transformed percent ratio of the restaurant industry (estimated degrees of freedom (EDF) = 3.24, *P* < 0.001), the Deprivation Index (DI) (EDF = 3.66, *P* = 0.015), and the proportion of essential workers (*β* =  − 0.10 (95% confidence interval − 0.15 to − 0.05, *P* < 0.001). We found a distinct decrease in subway ridership only in the least deprived areas, suggesting that social distancing is costly.

**Supplementary Information:**

The online version contains supplementary material available at 10.1007/s11524-021-00585-4.

## Introduction

Social distancing policy was implemented to prevent the spread of COVID-19 by reducing person-to-person contact [1]. However, studies have discussed the “luxury nature” [2] of social distancing (i.e., unequal impact on human mobility according to socioeconomic status) during the COVID-19 pandemic [3–5]. In the Republic of Korea, social distancing policies strongly relied on the voluntary participation of citizens without mandates [6] and exhibited short-term changes. In this situation, the effects of such policies varied depending on each community’s capacity to comply.

Many studies have analyzed human mobility patterns as a proxy for the effects of social distancing policy [3–5, 7]. Some of them focused on subway ridership [5, 8], because daily subway use for transportation is useful for examination of how daily movement patterns change with social distancing policies. Continued subway use during severe COVID-19 waves may indicate groups of people who were unable to work at home or change their transportation mode to a taxi or private car, despite the high-risk perception of the subway as a closed, crowded space [9]. The subway ridership pattern can be interpreted from a regional perspective, because it reflects the socioeconomic characteristics of the area around a subway station [10].

Here, we studied the unequal responses of communities to social distancing policy through subway use patterns, which reflected regional socioeconomic characteristics. We hypothesized that areas with higher socioeconomic status would show a greater decrease in subway use. We adjusted for the proportion of essential workers and for the leisure-purpose movements represented by restaurants in areas to explore the relationship.

## Methods

### Study Area

In Seoul, Korea, as of July 2021, there were nine major subway lines with 321 stations and a light line with 10 stations. We studied 294 stations on the nine major subway lines from January 1, 2020, to December 31, 2020. We aggregated the card tags of each station to the smallest administrative units of Seoul [11]. We treated stations on different lines differently; we used the mean value of a transfer station with several lines, considering heterogeneity in the amount of movement. Thus, 184 of the 425 administrative areas were included.

### Variables

The response variable was set as the percent change (%) in the reduction in subway travel between the period with the weaker social distancing level 1 (November 5 to 17, 2020) and the period with the stronger social distancing level 2.5 (December 10 to 22, 2020) (Appendix 1). We compared changes between the two periods to assess community adoption of rapidly strengthened policy. The percent change in subway ridership was averaged weekly to adjust for differences between weekdays and weekends.

The Deprivation Index (DI), density of restaurant industries, and proportion of essential workers were used as explanatory variables. To estimate the socioeconomic status of each region, we used the DI reported by the Seoul Health Foundation in 2018 [12], which was calculated using the Korean version of the deprivation index (KorDep_2015) developed by Kim et al. [13]. The DI was calculated by summing four standardized scores (z-scores) regarding the following proportions: households with automobiles, the population with less than a high school education, the population corresponding to the lower social class, and the divorced or widowed population.

The percentage of essential workers in each region was calculated as the sum of the numbers of workers engaged in agriculture, forestry, fishing, manufacturing, mining, water, electricity and gas, construction, passenger transportation, wholesale and retail, education, and healthcare, divided by the total number of workers in 2018. The restaurant industry variable was calculated as the log-transformed percentage of restaurant businesses (cafes, restaurants, bakeries, and pubs), compared with the total number of businesses in each region of 2018.

### Statistical Analysis

A generalized additive model (GAM) integrates a generalized linear model and additive model; it flexibly reflects nonlinear associations between response and explanatory variables [14, 15]. We used a GAM to make inferences concerning the mean percent change of the reduction in subway use (*y*) caused by the additive effects of socioeconomic factors in each region, and the smooth functions of each variable as:

$$y=\beta_0+\beta_1\times\mathrm{essential}\;\mathrm{workers}+\mathrm s\;\left(\log\;(\mathrm{restaurants})\right)+s\left(\mathrm{DI}\right)+\epsilon$$

Statistical analyses using GAMs were conducted using the “mgcv” package (ver. 1.8–28) in R ver. 4.0.4. A *p*-value < 0.05 from a two-sided statistical test was considered statistically significant.

## Results

**Fig. 1:**
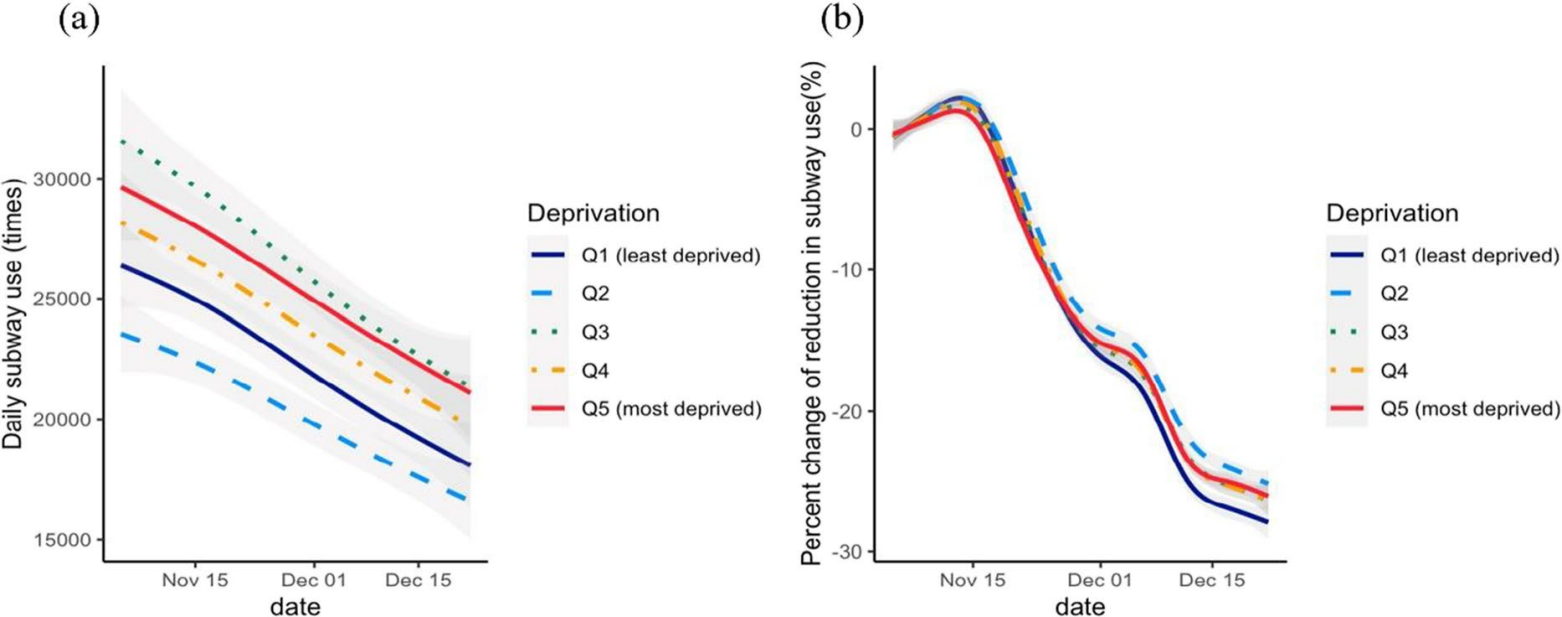
Changes in subway ridership with socioeconomic deprivation. GAM-smoothed lines with the 95% confidence intervals of the **a** weekly averaged subway use and **b** percent change in the weekly averaged subway use from November 5 to December 22, 2020, compared with baseline. The DIs of the study areas were classified in quintiles; higher quintiles indicate greater deprivation

Figure 1 shows that the subway use decreased during the period when the social distancing policy was strengthened quickly, from November 5, 2020 to December 22, 2020. During this period, subways were used more in deprived areas (Q3, Q4, and Q5) than in less deprived areas (Q1 and Q2). Using the percent change of the reduction (Fig. 1b), we found similar percent changes in areas Q3, Q4, and Q5; the reduction in subway use was approximately 30% greater in the least deprived area (Q1). Areas in Q2 had the least subway use, but the reductions in these areas were also smallest during that period.

**Fig. 2:**
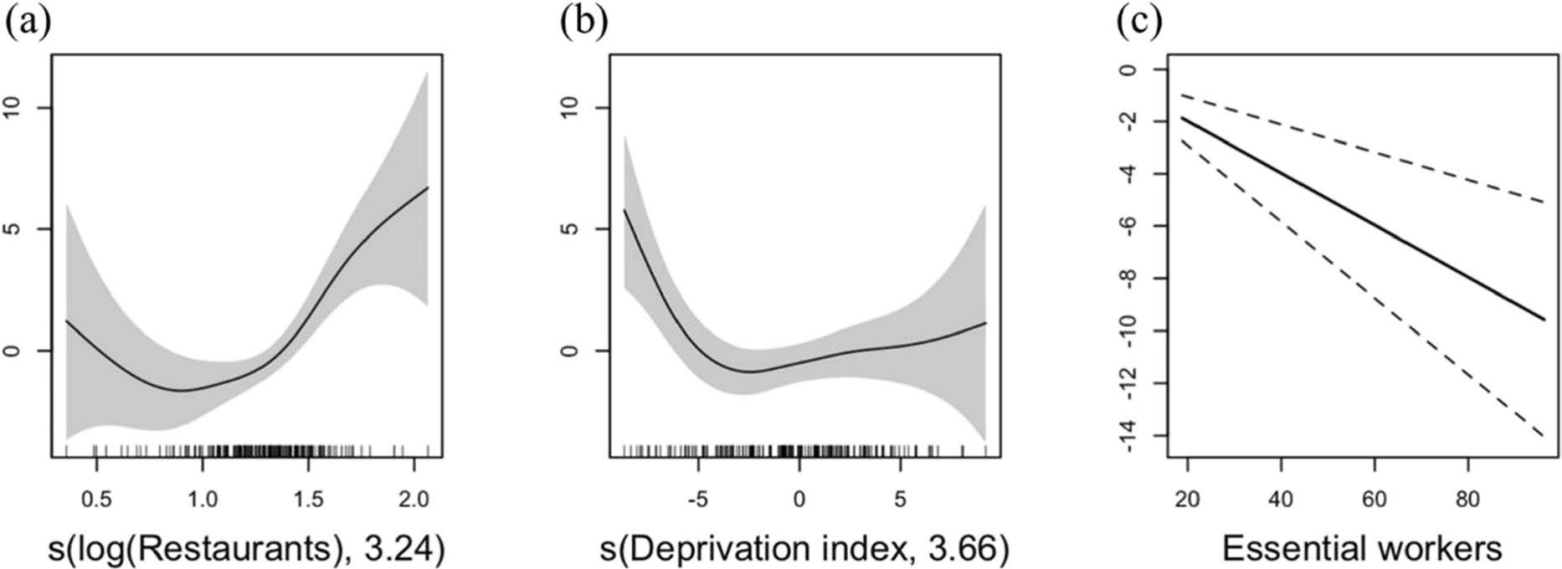
Effects of socioeconomic socioeconomic characteristic variable on the percent change in the reduction in subway ridership

We constructed a multivariate GAM model that included DI with other socioeconomic covariates (Fig. 2). The log-transformed restaurant industry (Estimated Degrees of Freedom (EDF) = 3.24, *P* < 0.001, Fig. 2a) and DI (EDF = 3.66, *P* = 0.015, Fig. 2b) had significant nonlinear associations with the changes in subway ridership. In comparison, the percentage of essential workers had a linear association with the percent change of subway ridership (*β* =  − 0.10 (95% confidence interval − 0.15 to − 0.05, *P* < 0.001, Fig. 2c). Despite adjustment for the negative effect of essential workers on the percent change of subway ridership, areas where restaurants contributed less than approximately 10% of businesses were negatively associated with the fitted values. Conversely, the percent change in subway ridership reduction was greater in areas where the percent ratio exceeded approximately 30% (Fig. 2a). The relationship in which less deprived areas had greater percent changes in subway ridership was evident only in areas with DI <  − 5 (Fig. 2b).

## Discussion

This study examined the unequal responses of communities to social distancing policy through differences in the reduction of subway usage by area. Despite adjustment for the effects of essential workers, the DI of a region and density of restaurants significantly contributed to a positive mean percent change in subway ridership. However, these contributions were evident only during a particular interval.

A large percent change in subway ridership was found in areas with a high proportion of restaurants, which indicated that such areas were more commercial in nature. Because highly commercial areas are usually clustered [16], people may avoid visiting such areas because of the risk-perception of crowded places. In comparison, the percent change in subway ridership decreased slightly in areas with a low proportion of restaurants. It is possible that administrative or other essential businesses are present in these areas.

The least deprived areas had the largest percent change in subway ridership. Similar results were observed in studies exploring the association between socioeconomic deprivation and mobility using mobile phone data in Spain and New Zealand. In both studies, mobility levels in low deprivation areas declined strongly during the lockdown period, then normalized more quickly [3, 7]. Our study is unique in that the association between socioeconomic deprivation and the percent change in subway ridership persisted, despite adjustment for the percentage of essential workers. This suggests that regardless of functional differentiation between essential and non-essential industries in assessed areas, the socioeconomic statuses of the regions caused differences in subway usage patterns.

An important finding was the “threshold effect” in social distancing; a significant reduction in subway ridership was found only in the least deprived areas. This suggests that people below a particular socioeconomic level perceive the costs of social distancing to outweigh their benefits because of economic insecurity [4, 17, 18]. According to Lou et al. [17], additional economic support and employment retention services for vulnerable low-income groups are needed, because structural employment factors may disproportionally affect mobility. The COVID-19 Marmot review [19] also recommended that employment-retention policies, such as the Coronavirus Job Retention Scheme, be extended for low-income workers; subsidies are recommended for firms and dismissed workers as direct and short-term strategies.

Furthermore, there is a need to build a broader, more robust safety net; our findings are consistent with the previous implication that intermediate socioeconomic groups experience a similar economic dilemma when following social distancing policies [19]. The authors of the previous study proposed methods to reduce precarious employment and invest in both quality and active labor market policies [19]. Our results suggest that financial support for more deprived communities is needed to enhance their capacities to provide various social services; support can also enhance the recovery of affected local businesses and employment. The ultimate goals of a community’s COVID-19 mitigation strategy, including social distancing, are to enable the community to thrive socially and economically by minimizing the adverse impact of the pandemic, and to end the pandemic by eventually bringing its spread under effective control [20].

Two limitations should be noted when interpreting our results. First, we used aggregated data that cannot explain individual patterns. Second, the age structures of the regions were not considered. Age variations in the proportion of the economically active population may affect mobility patterns. However, regions would have been self-controlled for age differences by determining the percentages between the two periods.

## Conclusion

We found different regional responses to social distancing policies arising from their socioeconomic characteristics. A distinct decrease in subway ridership was found only in the least deprived areas. This suggests the “luxury nature” of social distancing in Seoul. We propose that the economic security of citizens be prioritized to devise equal integrated responses to the epidemic.

## Supplementary Information

Below is the link to the electronic supplementary material.

Supplementary file1 (DOCX 6.14 MB)
