# Supplementary material for: The Unequal Effects of Social Distancing Policy on Subway Ridership during the COVID-19 Pandemic in Seoul, South Korea
Source: J Urban Health. 2022 Jan 1;99(1):77–81. doi: 10.1007/s11524-021-00585-4 (PMC8720159; doi:10.1007/s11524-021-00585-4)
Supplement: Supplementary file 1 — Supplementary file1 (DOCX 6.14 MB) [file 11524_2021_585_MOESM1_ESM.docx]

# Appendices


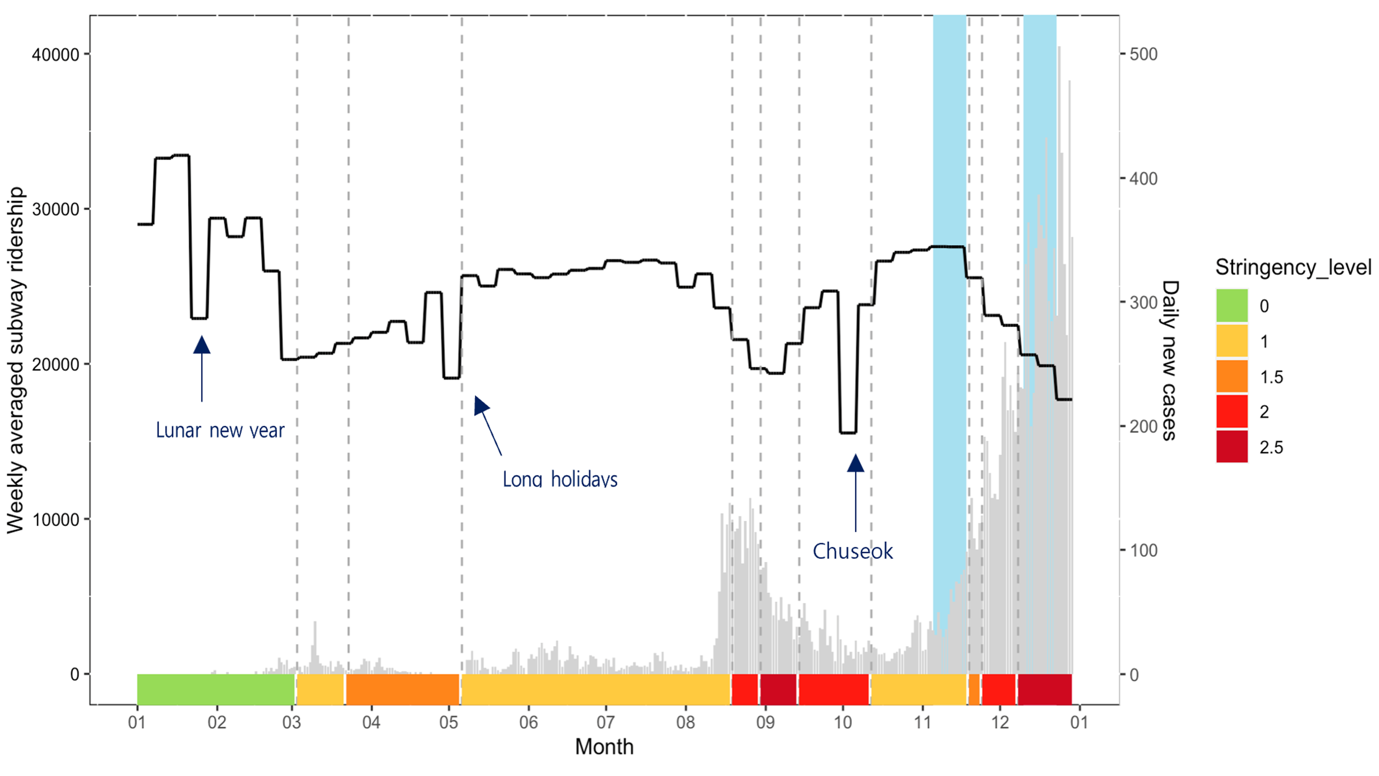


**A1** Changes in subway ridership according to the social distancing policy stringency level

Each plot shows the weekly averaged subway ridership according to the social distancing policy stringency level. There were five social distancing policy stringency levels (1, 1.5, 2, 2.5, and 3). At level ‘0’, no social distancing policy had yet been implemented. In 2020, the level of social distancing was not raised to its highest level (level 3). The dotted lines indicate the enforcement dates of the different social distancing policies. The percent change in subway usage between the two periods (in light blue) was analyzed (level 1, November 5–17, 2020; level 2.5, December 10–22, 2020). Footnote: ‘Long holidays’ include the consecutive holidays ‘Buddha’s Birthday’ (April 30), ‘May Day’ (May 1), weekend (May 2–3), and ‘Children’s Day (May 5), and ‘Chuseok’, which is Korean thanksgiving (one of the three major holidays in Korea)


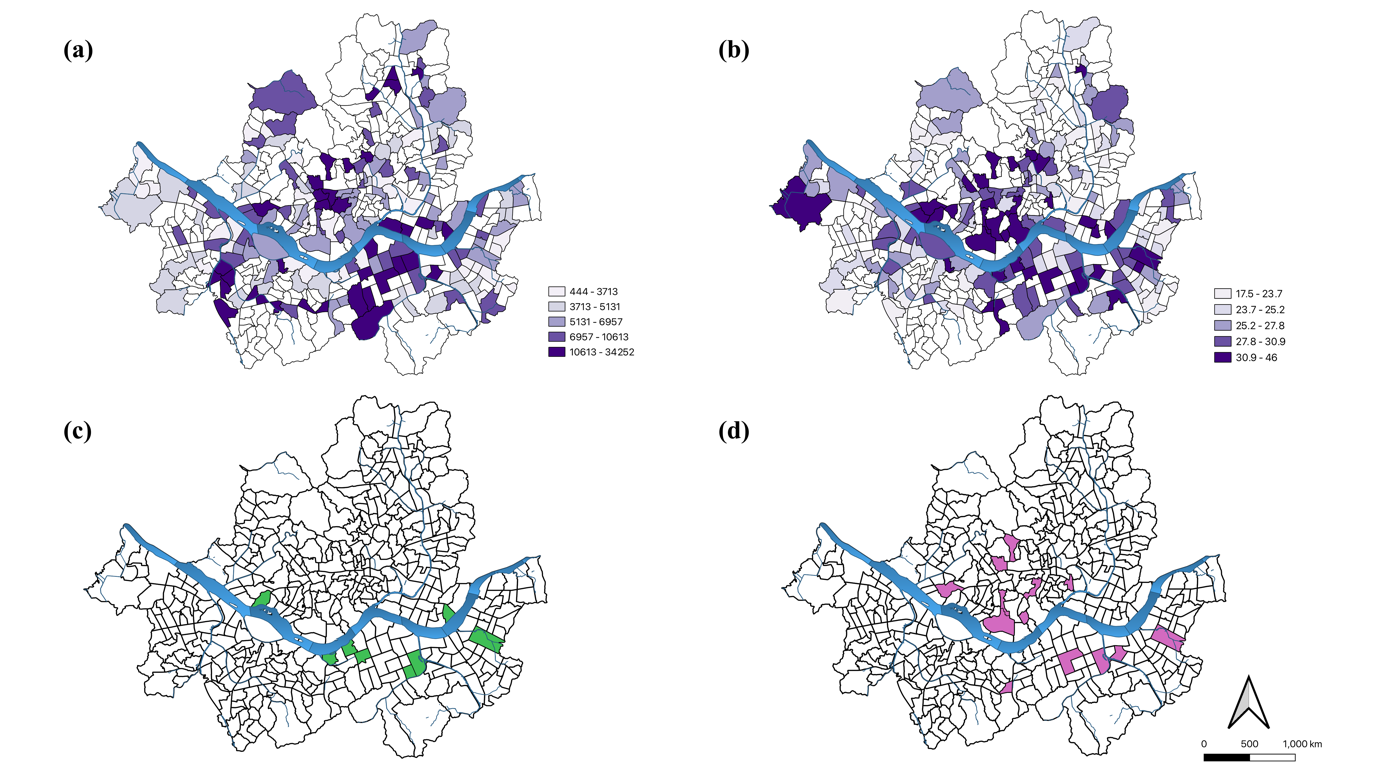


**A2** Changes in subway ridership and regional characteristics

Each map shows the changes in subway ridership when the stringency of social distancing policy was increased rapidly from the weaker level 1 to the stronger level 2.5 (level 1, Nov 5–17, 2020; level 2.5, Dec 10–22, 2020). Quintile maps of the (a) reduction in subway ridership and (b) percent change thereof. (c) The least-deprived areas had larger percent reductions in subway ridership (the highest was 20%). (d) Areas with the lowest proportion of essential workers had higher percent reductions in subway ridership (the highest was 20%)
